# In Utero Amniotic Fluid Stem Cell Therapy Protects Against Myelomeningocele via Spinal Cord Coverage and Hepatocyte Growth Factor Secretion

**DOI:** 10.1002/sctm.19-0002

**Published:** 2019-08-13

**Authors:** Yushi Abe, Daigo Ochiai, Hirotaka Masuda, Yu Sato, Toshimitsu Otani, Marie Fukutake, Satoru Ikenoue, Kei Miyakoshi, Hideyuki Okano, Mamoru Tanaka

**Affiliations:** ^1^ Department of Obstetrics & Gynecology Keio University School of Medicine Tokyo Japan; ^2^ Department of Physiology Keio University School of Medicine Tokyo Japan

**Keywords:** Amniotic fluid stem cells, Fetal cellular therapy, Hepatocyte growth factor, Myelomeningocele, Spinal cord

## Abstract

Despite the poor prognosis associated with myelomeningocele (MMC), the options for prenatal treatments are still limited. Recently, fetal cellular therapy has become a new option for treating birth defects, although the therapeutic effects and mechanisms associated with such treatments remain unclear. The use of human amniotic fluid stem cells (hAFSCs) is ideal with respect to immunoreactivity and cell propagation. The prenatal diagnosis of MMC during early stages of pregnancy could allow for the ex vivo proliferation and modulation of autologous hAFSCs for use in utero stem cell therapy. Therefore, we investigated the therapeutic effects and mechanisms of hAFSCs‐based treatment for fetal MMC. hAFSCs were isolated as CD117‐positive cells from the amniotic fluid of 15‐ to 17‐week pregnant women who underwent amniocentesis for prenatal diagnosis and consented to this study. Rat dams were exposed to retinoic acid to induce fetal MMC and were subsequently injected with hAFSCs in each amniotic cavity. We measured the exposed area of the spinal cord and hepatocyte growth factor (HGF) levels at the lesion. The exposed spinal area of the hAFSC‐treated group was significantly smaller than that of the control group. Immunohistochemical analysis demonstrated a reduction in neuronal damage such as neurodegeneration and astrogliosis in the hAFSC‐treated group. Additionally, in lesions of the hAFSC‐treated group, HGF expression was upregulated and HGF‐positive hAFSCs were identified, suggesting that these cells migrated to the lesion and secreted HGF to suppress neuronal damage and induce neurogenesis. Therefore, in utero hAFSC therapy could become a novel strategy for fetal MMC. stem cells translational medicine *2019;8:1170–1179*

**Significance Statement**

This study demonstrated that in utero amniotic fluid stem cell therapy for fetal myelomeningocele (MMC) could induce direct coverage of the spinal cord and hepatocyte growth factor secretion. These effects suppressed neural damage, such as neurodegeneration and astrogliosis, and promoted neural regeneration, which could improve the prognosis of MMC. Therefore, this therapy could become a novel strategy for fetal MMC.

## Introduction

Myelomeningocele (MMC) is a birth defect in which the vertebral column is open, and this condition is further complicated with spinal cord involvement during embryonic development. The exposed neural tissue degenerates in utero, resulting in sensorimotor dysfunction of the lower extremities, skeletal deformities, bladder and rectal disorders, and Chiari II malformations. Although the pathogenesis of MMC remains to be determined, the failure of neural tube closure leads to exposure of the spinal cord to the intrauterine environment (first hit) 1. If the neural tissue is not protected during pregnancy, the exposed spinal cord may be chemically and mechanically damaged and destroyed in utero until birth (second hit) 2. Thus, fetal MMC results in irreversible neurological impairments after birth. However, the options for prenatal treatment are still limited.

Interventions during pregnancy could protect the exposed spinal cord and prevent the second hit, but not the first hit, in theory. Fetal surgical repair of MMC has been found to improve the neurological outcomes for MMC fetuses compared with postnatal operation. However, interventions during pregnancy have a risk of maternal complications and preterm birth 3. To overcome these problems, in utero stem cell therapy could be a practical approach for the treatment of fetal MMC 4, 5, 6, 7, 8, 9, 10, 11, 12, 13.

Fetal cellular therapy is a treatment option for a variety of birth defects. The early prenatal diagnosis of MMC allows for the preparation of autologous human amniotic fluid stem cells (hAFSCs) for in utero stem cell therapy of affected fetuses, which could ameliorate long‐lasting, severe impairments, and deficits. Recently, several investigators demonstrated the therapeutic potential of the intra‐amniotic injection of mesenchymal stromal cells (MSCs) derived from the bone marrow, amniotic fluid, and embryonic stem cells (ESCs) using experimental models of MMC 4, 5, 6, 9, 14, 15, 16. However, the therapeutic effects on neuronal damage in the defective spinal cord and the mechanisms associated with in utero AFSC therapy are not well defined. In this study, we investigated the therapeutic effects of hAFSCs‐treatment on fetal MMC, and determined the mechanisms underlying these effects, using a rat model.

## Materials and Methods

### Isolation and Culture of hAFSCs

The study was approved by the institutional review board of Keio University School of Medicine (no. 20140285) and informed consent was obtained from all the volunteer donors. Five‐milliliter amniotic fluid samples were obtained from 15‐ to 17‐week‐old pregnant women who underwent amniocentesis. CD117‐positive (CD117^+^) cells were isolated as hAFSCs, as described previously 17, 18, 19, 20. Briefly, within 2 hours, cells were centrifuged at 200*g* for 5 minutes. After removing the supernatant, the cell pellet was cultivated in growth medium comprising alpha Modified Eagle Minimum Essential Medium (α‐MEM; Invitrogen, Carlsbad, CA), 15% fetal bovine serum (FBS; Invitrogen), 1% l‐glutamine (Invitrogen), 1% penicillin/streptomycin (Invitrogen), and 40% AmnioMax‐II (Life Technologies, Carlsbad, CA). After the cell population became subconfluent, these cells were stained with PE‐conjugated CD117 antibody and observed with a BZ‐X800 fluorescent microscope (KEYENCE, Osaka, Japan) to count the number of positive cells. CD117^+^ cells were isolated as hAFSCs using a Magnetic cell sorting kit (Miltenyi Biotec, Auburn, CA).

### Evaluation of Surface Marker Expression in hAFSCs

To evaluate the expression of surface markers on hASFCs, flow cytometry was performed as described in our previous studies 18, 20. A total of 1 × 10^5^ cells were harvested and incubated with either PE‐, FITC‐, or APC‐conjugated antibodies at 4°C for 1 hour and with appropriate isotype controls. Stained cells were then analyzed using a MoFlo XDP flow cytometer (Beckman Coulter, Brea, CA) using Cell Quest software (Becton Dickinson and Company, Franklin Lakes, NJ), and data were analyzed using Kaluza software (Becton Dickinson and Company). Antibody information is listed in Supporting Information Table S1.

### Differentiation Potential of hAFSCs

To investigate the differentiation ability, hAFSCs were differentiated in vitro into osteogenic, adipogenic, and chondrogenic lineages. hAFSCs were independently cultured either in “adipogenic differentiation medium,” “osteogenic differentiation medium,” or “chondrogenic differentiation medium” (Lonza, Basel, Switzerland) at 37°C in 5% CO_2_ for the appropriate time according to the manufacturer's recommended protocol. Osteogenesis was assessed by Alizarin staining (Cosmo Bio Co., Ltd., Tokyo, Japan) of the calcified extracellular matrix deposition. Oil Red O staining was used to detect intracellular lipid droplet formation to evaluate adipogenesis. Chondrogenic differentiation was determined by Alcian blue staining.

### Retinoic Acid‐Induced Rat MMC Model

All experimental protocols were approved by the Institutional Animal Care at Keio University (approval number 16079‐[0]). Fetal rats with MMC were created based on a protocol described previously 2, 21. Briefly, Sprague‐Dawley rats (Charles River Laboratories Japan, Inc., Kanagawa, Japan) were used for this study. After brief exposure to isoflurane (Abbott Laboratory, Chicago, IL), pregnant rats were orally administered 60 mg/kg of retinoic acid (RA; Sigma–Aldrich, St. Louis, MO) dissolved in olive oil (10 mg/kg) at embryonic day 10 (E10). To determine the optimal timing of RA administration to induce a high incidence of isolated MMC‐like defects in fetal rats, pregnant dams were gavage‐fed single doses of RA on E10 at four time points: 0:00 a.m. (group 1), 6:00 a.m. (group 2), 0:00 p.m. (group 3), and 6:00 p.m. (group 4).

### Intra‐Amniotic Injection of hAFSCs

The dams received an injection of hAFSCs into the intra‐amniotic cavity on E17. General anesthesia was induced and maintained in a chamber with isoflurane (Abbot Lab), and was inhaled at 2%–4% in 100% oxygen. A midline incision was made and the bicornuate uterus was exposed. A 32‐G disposable needle (Dentronics Co., Ltd., Tokyo, Japan) on a 100‐μl syringe (Hamilton Company, Reno, NV) was introduced into every amniotic cavity containing a viable fetus via the ventral aspect of the fetus, carefully avoiding the fetus, placenta, and umbilical cord (Supporting Information Fig. S1A). Each injection consisted of either hAFSCs (1 × 10^5^ cells per fetus) suspended in 50 μl phosphate‐buffered saline (PBS) or 50 μl of PBS only (as a control); hAFSCs or control inoculations were alternately injected from the left‐most fetus toward the right fetus (Supporting Information Fig. S1A). The uterus was then returned to the abdomen and the incision was closed in two layers with 3‐0 Vicryl and 4‐0 Vicryl (Ethicon, Inc., Somerville, NJ) simple running locking sutures.

### Macroscopic Analysis

All rat dams were euthanized under isoflurane anesthesia on E21. Fetuses were examined for the presence or absence of external abnormalities including MMC, and the numbers of fetuses with external abnormalities were recorded (Supporting Information Fig. S1B and Table 1). Only fetuses with isolated MMC in group 2 (Supporting Information Fig. S1C) were used for further analysis, because fetuses with isolated MMC after RA exposure were most abundant in this group. The overall fetal crown–rump length, as well as the longest cranial–caudal and lateral dimensions of the spina bifida defects, was measured using digital Vernier calipers (A&D Company, Tokyo, Japan).

**Table 1:** Phenotypic characterization of retinoic acid‐induced rat myelomeningocele model

Time point	Group 1	Group 2	Group 3	Group 4
Fetal death	100.0	12.9	0.0	0.0
Sirenomelia	0.0	3.2	0.0	0.0
Myelomeningocele	0.0	83.9	8.0	0.0
Spina bifida occulta	0.0	0.0	60.0	7.1
No lesions	0.0	0.0	32.0	92.9
Total	100	100	100	100

### Histological Analysis

To evaluate the therapeutic effects and mechanisms of in utero AFSC therapy on the exposed spinal cord, the cross‐sectional areas of the defective spinal cords (spinal x‐section) were histologically analyzed at the level of the maximum transverse diameter of the exposed lesion. Excised specimens were fixed with 4% paraformaldehyde for paraffin embedding. Paraffin sections (4‐μm) were then stained with H&E. Furthermore, neurons and astrocytes were evaluated by staining spinal x‐sections with anti‐Tubulin‐βIII (Abcam, Cambridge, MA) and anti‐glial fibrillary acidic protein (GFAP) antibodies (Dako Corporation, Carpinteria, CA), respectively 2. Additionally, human derived‐cells were visualized using the human‐specific antigen STEM121 (Cellartis–Takara Bio, Kusatsu, Japan, Y40410), which was used to immunostain spinal x‐sections. To examine the effect of hAFSCs, immunostaining with antibroad‐spectrum cytokeratin (Abcam), anti‐CXCL12 (stromal cell‐derived factor 1; Santa Cruz Biotechnology Inc., Santa Cruz, CA), antihepatocyte growth factor (HGF; Abcam), anti‐c‐Met (Bioss Antibodies, Woburn, MA), and anti‐p‐Met antibodies (R&D Systems, Minneapolis, MN) was performed. Antibody information for immunostaining is provided in Supporting Information Table S2. Stained sections were viewed using a Zeiss LSM710 confocal laser scanning microscope (Zeiss, Oberkochen, Germany) and morphometric analysis was performed using the obtained images and ImageJ software (www.rsb.info.nih.gov/ij).

### Analysis of E21‐Rat Amniotic Fluid Cells

Approximately 1 ml of rat amniotic fluid was collected per fetus. Samples were immediately centrifuged at 300*g* for 5 minutes. After removing the supernatant, the cell pellet was cultivated in growth medium consisting of α‐MEM (Invitrogen), 15% FBS (Invitrogen), 1% l‐glutamine (Invitrogen), 1% penicillin/streptomycin (Invitrogen), and 40% AmnioMax‐II (Life Technologies). Amniotic cells were plated for further analysis. To enumerate human‐derived cells in E21 rat amniotic fluid, STEM121 immunostaining was performed, and stained cells were viewed by LSM710 microscopy (Zeiss). Moreover, to determine the expression of trophic factors derived from hAFSCs in rat amniotic fluid, we isolated CD117^+^ amniotic fluid cells from E19‐ and E21‐rat amniotic fluid cells using CD117 MicroBeads (Miltenyi Biotec).

### RNA Isolation and Quantitative Real‐Time PCR

Total RNA was isolated using the RNeasy mini kit (Qiagen, Hilden, Germany) according to manufacturer's instruction. Reverse transcription of total RNA was performed using the Prime Script RT Master Mix (Takara Bio Inc., Shiga, Japan). Quantitative polymerase chain reaction (PCR) was performed in duplicate in a volume of 25 μl per reaction using a 96‐well Bio‐Rad CFX96 Real‐Time PCR System (Bio‐Rad, Richmond, CA). Reaction mixtures included 5 ng of genomic DNA as the template, 0.4 mM of each primer (Thermo Fisher Scientific, Waltham, MA), SYBR Premix Ex Taq II (Tli RNaseH Plus; Takara Bio), and sterile H_2_O 18, 20. The primer sets are listed in Supporting Information Table S3. These primers did not distinguish between the expression of rat‐derived and human‐derived transcripts. We analyzed the relative gene expression in each sample by the 2^−ΔΔCT^ method. Gene expression values were normalized to *GAPDH* levels as an internal control.

### Statistical Analysis

All results are expressed as the mean ± SD. Quantitative variables were statistically analyzed using a one‐way analysis of variance followed by a Student's *t* test. The ordinal variables were analyzed using a Mann–Whitney *U* test; *p* < .05 was considered statistically significant. Each analysis was performed with commercially available software, namely IBM SPSS Statistics Version 24 (IBM Corporation, Armonk, NY).

## Results

### Isolation and Characterization of hAFSCs

Before isolation, human amniotic fluid cells were stained with an anti‐CD117 antibody, and the CD117^+^ cells were counted. There were 317 human amniotic fluid cells in 10 random visual fields. Of these, 17 (5.4%) cells were positive for CD117 (Supporting Information Fig. S2A). In accordance with our previous studies 18, 20, spindle‐shaped CD117^+^ cells (i.e., hAFSCs) were expanded (Supporting Information Fig. S2B). These cells were positive for mesenchymal markers (CD19, CD44, CD73, CD90, and CD105) and negative for hematological markers (CD14, CD34, and human leukocyte antigen‐DR; Supporting Information Fig.R S2C). Moreover, these cells have a potential to differentiate toward adipogenic, osteogenic, and chondrogenic lineages (Supporting Information Fig. S2D–S2F).

### Determination of Optimal Timing of RA Administration

Overall, we examined 116 rat fetuses divided into four groups according to the time of administration of all‐trans RA. In group 1, all fetuses died. Among the fetuses in groups 2 and 3, isolated MMC was observed in 52 (83.9%), and two (8.0%) animals, respectively, and no fetus exhibited combined exencephaly and MMC. In group 2, two fetuses had sirenomelia sequence (3.2%; Supporting Information Fig. S1B, S1C and Table 1). Almost all fetuses in group 4 had no external malformations. Therefore, only fetuses with isolated MMC in group 2 were used for further analysis.

### hAFSCs‐Treatment Reduces Skin Defect Size and Protects the Exposed Spinal Cord

**Figure 1:**
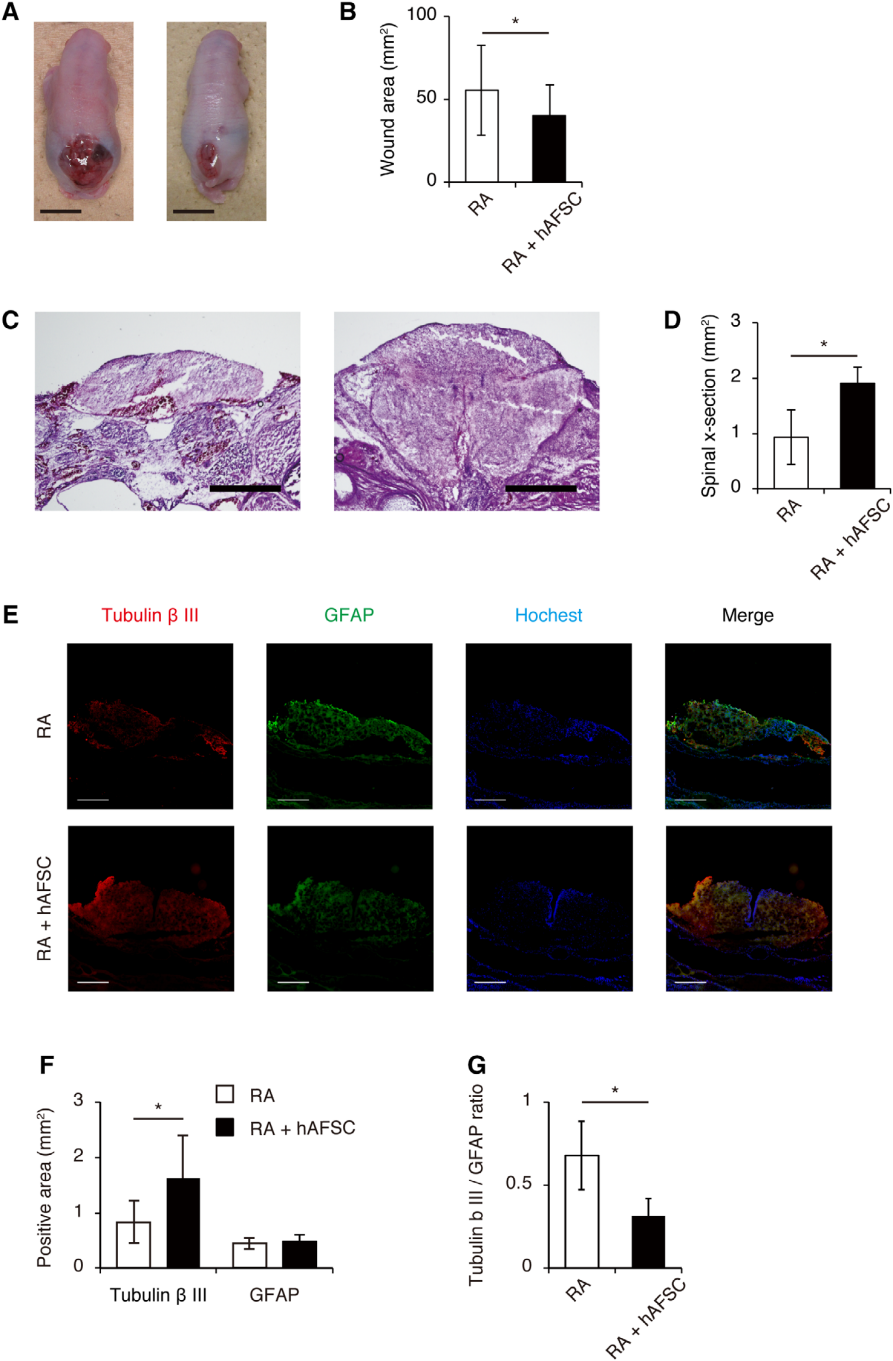
In utero human amniotic fluid stem cell (hAFSC) therapy reduces skin defect size and protects the exposed spinal cord. **(A):** Representative images of isolated myelomeningocele in retinoic acid (RA) group (left) and RA + hAFSC group (right). Scale bars: 500 μm. **(B):** Analysis of skin defect area (*n* = 5). **(C):** Representative images of H&E staining of spinal cross‐sections in RA group (left) and RA + hAFSC group (right). **(D):** Analysis of spinal cross‐section area (*n* = 5). **(E):** Representative images of Tubulin‐βIII and GFAP immunostaining of spinal cross‐sections in RA and RA + hAFSC group. **(F):** Analysis of Tubulin‐βIII‐positive area (*n* = 5), GFAP‐positive area (*n* = 5), and **(G)** GFAP/Tubulin‐βIII positive area ratio (*n* = 5). Images are representative of at least three independent experiments. Results are presented as mean ± SD; *, *p* < .05 compared with control.

There were no significant differences in fetal crown–rump lengths between the RA and RA + hAFSC groups. However, the cranial–caudal and lateral dimensions and MMC defect areas, both based on absolute values and adjusted for fetal crown–rump length, were significantly decreased in the RA + hAFSC group compared with those in the RA group (RA group versus RA + hAFSC group; 53.92 ± 26.29 mm^2^ versus 39.21 ± 18.03 mm^2^; *p* < .05; Fig. 1A, 1B, Table 2). To evaluate the protective effects of the intra‐amniotic injection of hAFSCs on the exposed spinal cord, spinal x‐sections were histologically analyzed at the level of the maximum transverse diameter of the exposed lesion. Histological analysis demonstrated a significant increase in the spinal x‐section (RA group vs. RA + hAFSC group; 0.95 ± 0.29 mm^2^ vs. 1.94 ± 0.50 mm^2^; *p* < .05; Fig. 1C, 1D). These results indicated that hAFSCs promote skin coverage of the cutaneous defect and protect the exposed spinal cord.

**Table 2:** Fetal crown–rump length, cranial–caudal dimensions, lateral dimensions, and areas of cutaneous defect

Group	CRL (mm)	MCC (mm)	MT (mm)	S (mm^2^)	MCC/CRL	MT/CRL	S/(CRL)^2^
RA	33.18 ± 3.10	5.34 ± 1.53	3.16 ± 1.45	53.92 ± 26.29	0.16 ± 0.05	0.10 ± 0.04	0.0245 ± 0.01
RA + hAFSC	32.71 ± 3.02	4.54 ± 1.90	2.99 ± 1.05	39.21 ± 18.03	0.14 ± 0.05	0.09 ± 0.03	0.0321 ± 0.02
Overall *t* test	0.61	1.8	0.51	2.6	1.8	0.51	1.92
Overall *p* value	.27	.04	.3	.01	.04	.3	.03

Abbreviations: CRL, crown‐rump length of the fetus; hAFSC, human amniotic fluid stem cell; MMC, cranio–caudal length of the myelomeningocele defect; MT, transverse length, or width of the myelomeningocele defect; RA, retinoic acid.

To evaluate neuronal cell damage in the defective spinal cords, Tubulin‐βIII staining was performed on spinal x‐sections, because it is indicative of neurons and is the earliest marker of neurogenesis 2, 22. The Tubulin‐βIII‐positive area was significantly increased after hAFSCs‐treatment (RA group vs. RA + hAFSC group; 0.81 ± 0.37 mm^2^ vs. 1.60 ± 0.75 mm^2^; *p* < .05; Fig. 1F). Moreover, double immunohistochemistry for Tubulin‐βIII and GFAP was performed to investigate astrogliosis in the defective spinal cords (Fig. 1E) 2, 22. A decline in the ratios of GFAP‐ to Tubulin‐βIII‐positive areas after hAFSC‐treatment showed that hAFSCs could reduce astrogliosis (RA group vs. RA + hAFSC group; 0.68 ± 0.40 vs. 0.32 ± 0.20; *p* < .05; Fig. 1G). These results suggested that hAFSCs protected neuronal cells in the exposed spinal cord by reducing astrogliosis and inducing neurogenesis in the lesion.

### hAFSCs‐Treatment Reduces Inflammatory Reactions in the Exposed Spinal Cord

**Figure 2:**
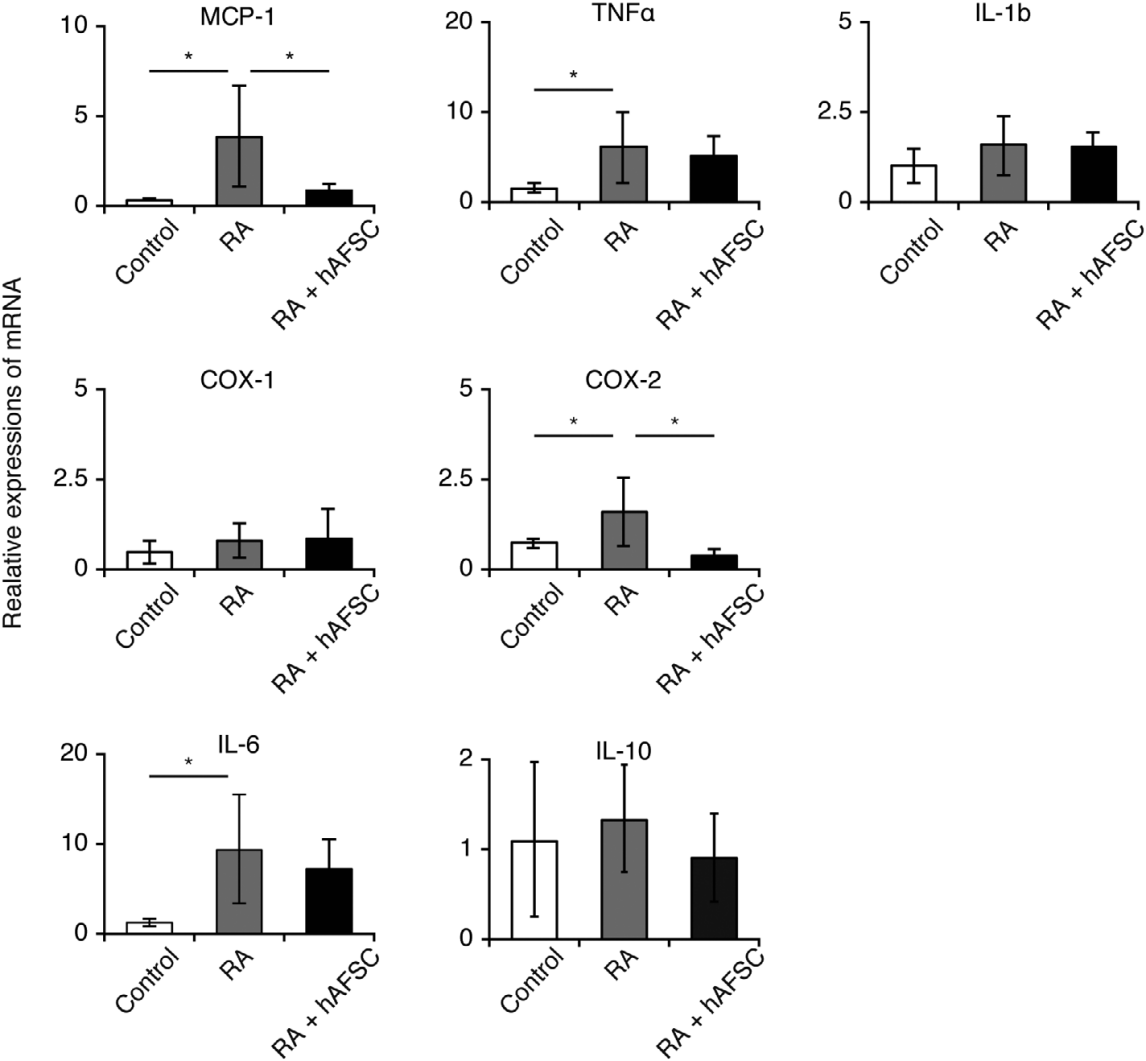
Human amniotic fluid stem cells (hAFSCs)‐treatment increases hepatocyte growth factor production and reduces inflammatory reactions in exposed spinal cord. Retinoic acid was used to induce myelomeningocele in rats and hAFSCs‐treatment was performed. RT‐qPCR analysis of pro‐ or anti‐inflammatory cytokine levels in the exposed spinal cord. Results are presented as mean ± SD; *, *p* < .05 compared with control.

Inflammation caused by amniotic fluid exacerbates spinal cord lesions during fetal MMC and is likely to act as a “second hit.” To determine the therapeutic effect of hAFSCs‐treatment on inflammatory responses in the spinal cord, the mRNA expression of several inflammatory mediators was analyzed. Among them, RA induction significantly increased *monocyte chemoattractant protein–1 (MCP‐1)*, *interleukin (IL‐6)*, *tumor necrosis factor alpha (TNF‐α)*, and *cyclooxygenase (COX‐2)* mRNA levels, and these responses tended to be attenuated by hAFSCs‐treatment, especially for *MCP‐1* and *COX‐2* (Fig. 2).

### hAFSCs‐Treatment Reduces Neuronal Damage in the Muscular Layer of the Bladder

Previous reports suggested that Tubulin‐βIII expression in the bladder muscular layer was reduced by RA exposure and restored at E17 by in utero repair using a chitosan‐gelatin membrane patch 23, 24, 25. In the present study, both RA and RA + hAFSC bladders had well‐organized smooth muscle bundles and there were no differences in α‐SMA expression patterns between the two group (Supporting Information Fig. S3A, S3B). However, Tubulin‐βIII‐positive thin nerve fibers were restored by hAFSCs‐treatment (Supporting Information Fig. S3C). These results indicated that hAFSCs‐treatment could reduce neuronal damage in the muscular layer of the bladder.

### hAFSCs Engraft onto the Surface of the Defective Spinal Cord via CXCL12 Signaling and Differentiate or Produce HGF

**Figure 3:**
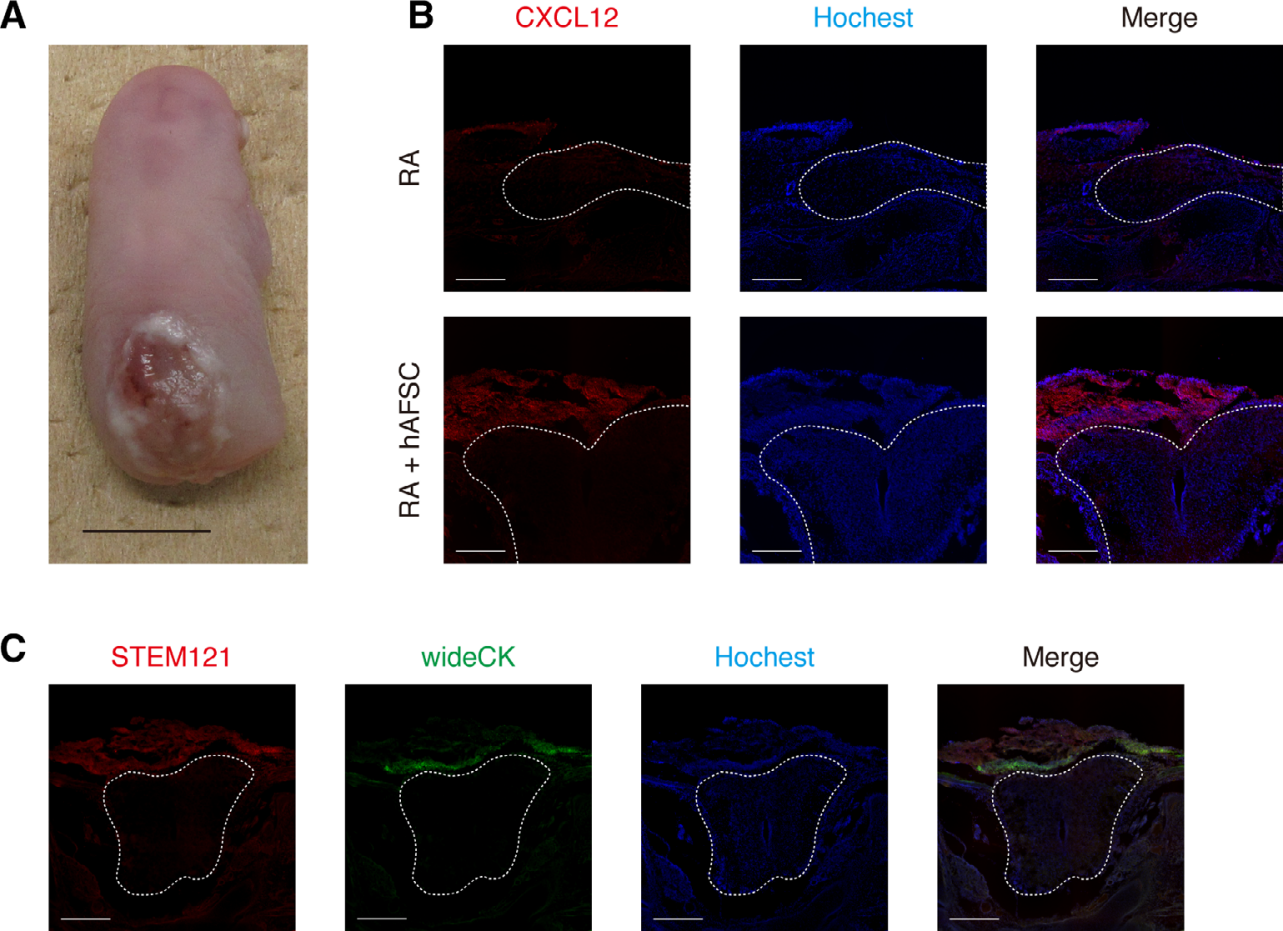
Human amniotic fluid stem cells (hAFSCs) engraft onto the surface of the defective spinal cord via CXCL12 signaling and differentiate into cytokeratin‐expressing cells. Retinoic acid (RA) was used to induce myelomeningocele in rats and hAFSCs‐treatment was performed. **(A):** Representative image of the response to hAFSCs‐treatment (scale bars: 500 μm). **(B):** Representative images of spinal cross‐sections stained with CXCL12 (×40; scale bars: 500 μm) in the RA and RA + hAFSC group. **(C):** Representative images of spinal cross‐sections stained with STEM121 and broad‐spectrum cytokeratin antibodies (×40; scale bars: 500 μm).

Several investigators have reported that cells preferentially home to the exposed spinal cord using a rat fetal MMC model 4, 6. However, the role of hAFSCs engrafted on the surface of the defective spinal cord remains to be determined 26. Interestingly, rudimentary skin coverage was macroscopically distinguishable in this study (Fig. 3A). Chemokines including CXCL12 and CXCR4 play a crucial role in the recruitment of MSCs to lesion sites 27. After fetal MMC induction, CXCL12 expression was slightly increased on the surface of the spinal cord and was markedly increased by hAFSCs‐treatment. Moreover, CXCL12 was mainly observed in human‐derived cells (Fig. 3B). These results indicated that hAFSCs accumulate on the surface of the defective spinal cord via CXCL12 signaling, which is mainly derived from hAFSCs that were engrafted on the lesion. We also showed that some cells on the defective spinal cord were double‐positive for STEM121 and cytokeratin, which is a main component of the skin epidermis (Fig. 3C), whereas the other cells that were positive for STEM121 were negative for this marker.

**Figure 4:**
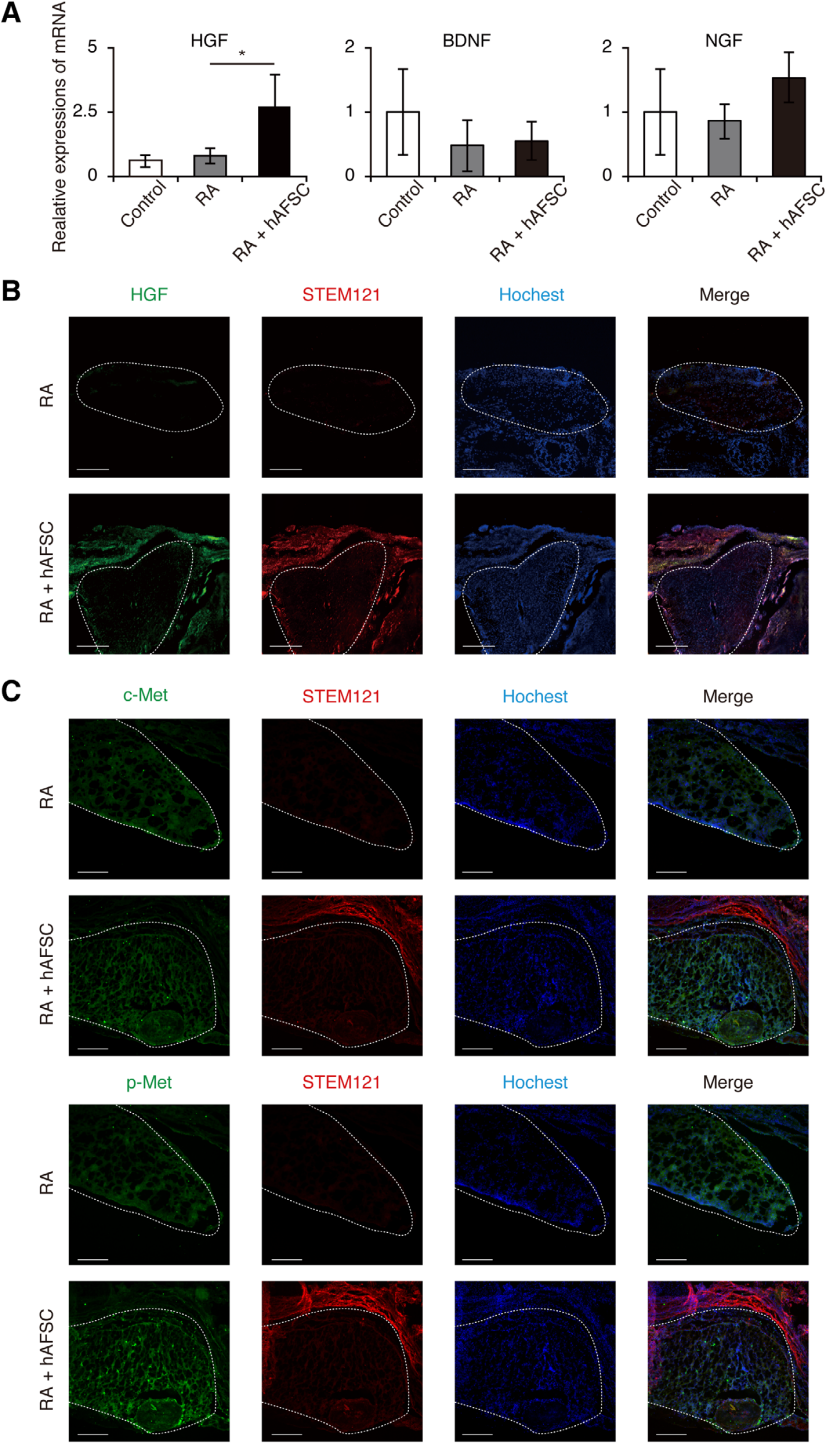
Engrafted human amniotic fluid stem cells (hAFSCs) produce hepatocyte growth factor (HGF). Retinoic acid (RA) was used to induce myelomeningocele in rats and hAFSCs‐treatment was performed. **(A):** RT‐qPCR analysis of growth factor levels in the exposed spinal cord. Results are presented as mean ± SD; *, *p* < .05 compared with control. **(B):** Representative images of STEM121 and HGF double staining (×40; scale bars: 500 μm). **(C):** Representative images of c‐Met and phosphorylated c‐Met staining of spinal cross‐sections in RA group and RA + hAFSC group (×40; scale bars: 500 μm). Images are representative of at least three independent experiments.

Next, we hypothesized that paracrine mediators could play an important role in the protection against spinal cord damage; accordingly, we determined the expression of growth factors in the spinal cord that are known as key molecules involved in spinal cord protection. Among them, we found that only *HGF* mRNA was significantly elevated upon hAFSCs‐treatment (Fig. 4A). Moreover, immunofluorescence analysis clarified that some cells were double‐positive for STEM121 and HGF (Fig. 4B). Furthermore, p‐Met, the phosphorylated HGF receptor, was upregulated in the RA + hAFSC group compared with the levels in controls (Fig. 4C).

These data indicated that neoepidermal cells directly differentiate from hAFSCs to protect the defective spinal cord from chemical and mechanical injury in utero. In contrast, undifferentiated hAFSCs produce HGF, which contributes to the suppression of inflammatory reactions and the promotion of neural protection and regeneration in a paracrine manner.

### hAFSCs Integrate into Rat Amniotic Fluid and Secrete HGF

**Figure 5:**
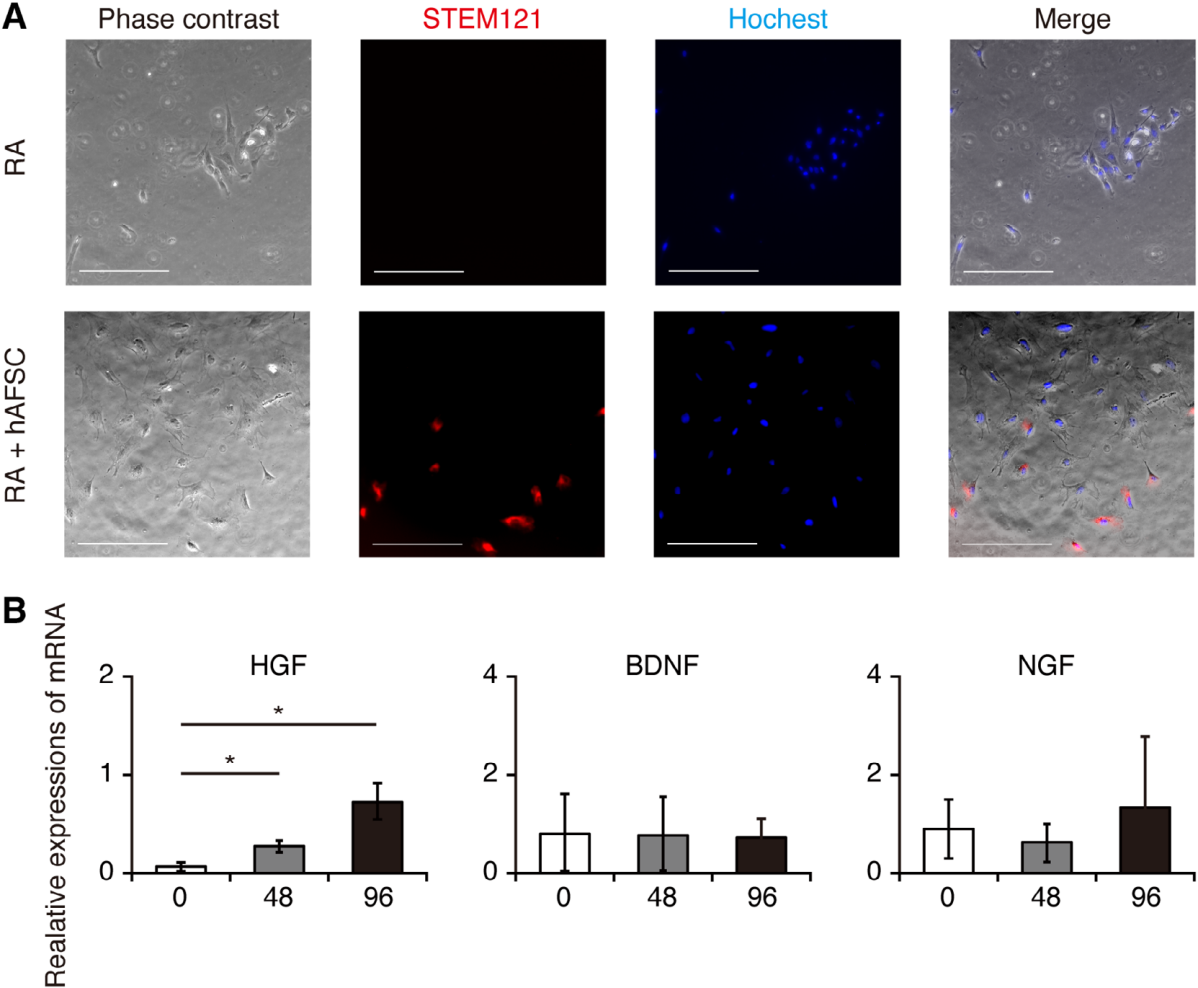
Human amniotic fluid stem cells (hAFSCs) integrate into myelomeningocele–rat amniotic fluid and produce hepatocyte growth factor. **(A, B):** Representative images of E21‐rat amniotic fluid cells (A) under phase contrast microscopy and STEM121 staining (×40; scale bars: 200 μm). (B): RT‐qPCR analysis of paracrine mediators in CD117 positive (CD117^+^) amniotic fluid cells obtained from E19 (48 hours after hAFSCs injection) and E21 (96 hours after hAFSCs injection) rat amniotic fluid cells compared with those from hAFSCs before injection into rat amniotic fluid. Results are presented as mean ± SD; *, *p* < .05 compared with control.

All hAFSCs injected into the rat amniotic fluid did not adhere to the fetal MMC lesion. To investigate the role of these cells, amniotic fluid cells collected from E21 rat amniotic fluid were plated. Immunocytochemical analysis for the human‐specific marker STEM121 was performed. In the RA + hAFSC group, STEM121‐positive cells and negative cells were observed. In contrast, only STEM121‐negative amniotic fluid cells were visible in the RA group (Fig. 5A).

Almost all CD117^+^ cells obtained from E19‐ and E21‐amniotic fluid were positive for STEM121, suggesting that these cells originated from hAFSCs injected at E17 (data not shown). To identify the molecular mediators that are secreted from xenografted hAFSCs in rat amniotic fluid, we analyzed mRNA levels of paracrine mediators in CD117^+^ amniotic fluid cells obtained from E17 (before hAFSCs injection), E19 (48 hours after hAFSCs injection), and E21 (96 hours after hAFSCs injection) rat amniotic fluid cells. We found that among paracrine mediators examined in this study, only *HGF* mRNA expression was significantly increased after hAFSCs‐treatment, and this occurred in a time‐dependent manner (Fig. 5B). These results suggested that hAFSCs injected into E17‐rat amniotic fluid integrated into the rat amniotic fluid and produced HGF.

## Discussion

hAFSCs, injected into the amniotic fluid as fetal stem cell therapy, have been shown to preferentially home to the exposed spinal cord using rat fetal MMC models 4, 6. However, the biological basis for hAFSCs‐treatment remains to be elucidated 26. To the best of our knowledge, this is the first study to demonstrate the therapeutic mechanisms of intra‐amniotic hAFSCs‐injection for the treatment of fetal MMC. In this study, we found that hAFSCs exerted their effect on fetal MMC via two different mechanisms, specifically direct coverage of the spinal cord lesion and HGF secretion. Finally, this treatment reduced neuronal damage such as neurodegeneration and astrogliosis and promoted neural regeneration.

hAFSCs migration to the spinal cord lesion after injection into the amniotic cavity is considered the first step for treatment. Chemokines secreted by inflammatory cells such as monocytes and T lymphocytes play a crucial role in the migration of MSCs to the site of inflammation 28. hAFSCs were found to engraft onto the surface of the defective spinal cord, at least in part, via endogenous CXCL12/CXCR4 signaling after spinal cord damage induced by RA exposure. Next, hAFSCs on the lesion produced human‐derived CXCL12 and contributed to the further accumulation of hAFSCs in amniotic fluid. hAFSCs have the potential to migrate to the lesion via CXCL12/CXCR4 signaling, based on the results from several rodent models such as bleomycin‐induced pulmonary fibrosis 29, renal intestinal fibrosis 30, and a sciatic nerve injury 31; thus, in our study, hAFSCs injected into the uterine cavity might accumulate in the lesion due to concentration gradients of CXCL12 that originate from the exposed spinal cord.

The reduction in the MMC defect size could lead to spinal cord protection by attenuating the “second hit.” In this study, we clearly demonstrated that hAFSCs can cover the MMC defect and that some hAFSCs directly differentiate into cytokeratin‐expressing cells, a main component of the skin epidermis. Previously, we and other groups reported that hAFSCs themselves and their secretomes can accelerate wound closure by enhancing re‐epithelialization using a dorsal excisional cutaneous wound model in BALB/c mice 20, 32, 33. Furthermore, hAFSCs promote cutaneous wound closure through the direct differentiation into keratinocytes in vivo and have the potential to differentiate into epidermal‐lineage cells including keratinocytes of various maturity levels in vitro 32. Based on these findings, our data indicated that hAFSCs attach to the surface of the spinal cord and cover the lesion with neoepidermal cells that directly differentiate from hAFSCs and promote epidermal ingrowth stimulated by some hAFSCs‐derived paracrine mediators, leading to protection from the “second hit” during pregnancy.

In addition to the observed coverage of the MMC defect, HGF secreted from hAFSCs could play a crucial role in reducing neuronal damage in a paracrine manner. The principal therapeutic mechanism of MSC therapy has been considered the release of trophic factors. However, these secreted trophic factors differ depending on the MSC origin, culture conditions, preconditioning by hypoxia, inflammation, drugs, and the environment surrounding the engrafted MSCs 34, 35. AFSCs have been reported to improve and restore cellular function in the injured nervous system by reducing the inflammatory response, stimulating endogenous repair through the recruitment of progenitor cells, and promoting neuronal outgrowth. This was shown using sciatic nerve injury and stroke models 31, 36, 37, 38, as well as our data using a neonatal hypoxic–ischemic encephalopathy model 18. However, key paracrine factors that are secreted from AFSCs remain controversial based on these studies. In the present study, for the first time, we identified that HGF is a key neurotrophic mediator that is secreted from both hAFSCs engrafted on the defective spinal cord and those in the amniotic fluid using an RA‐induced model of rat MMC.

HGF was first identified as a potent mitogen for mature hepatocytes 39, 40 and a natural ligand for the c‐Met receptor 41. Recent studies revealed that HGF acts as a neurotrophic factor for a variety of neuron types, enhances angiogenesis, reduces inflammation, improves microcirculation, and exerts a neuroprotective effect during a variety of neuronal disease models such as cerebral ischemia 42, amyotrophic lateral sclerosis 43, and spinal cord injury 44, 45, 46. In the present study, HGF reduced inflammatory reactions including astrogliosis and protected neural elements from the “second hit.” In addition, we used Tubulin‐βIII as an indicator to evaluate the degree of neural differentiation and the cytoarchitecture of MMC. Given that Tubulin‐βIII is the earliest marker of neurogenesis 2, 22, hAFSCs‐treatment was also found to induce neurogenesis in fetal MMC via HGF secretion.

Previous reports have suggested that hAFSCs could be easily isolated and expanded, and have the ability to differentiate into various cell types, reduce the inflammatory response, and stimulate endogenous repair without any ethical concerns and the risk of tumorigenesis 17, 19. Preparation of an adequate number of autologous hAFSCs for treating MMC fetuses requires only a small number of amniotic fluid cells collected by amniocentesis; this is associated with minimal invasive risk for the patient. Based on results of the present study, hAFSCs offer intriguing potential for autologous hAFSCs‐treatment for MMC fetuses 13. However, there are several limitations to this research. Serious complications of MMC such as Chiari malformation and bladder/rectal disorders remain to be determined and the most important problem is that RA‐induced MMC rats die immediately after birth, and thus, the long‐term outcomes with respect to motor dysfunction cannot be studied. Therefore, we would like to address this question using additional in vivo studies like a surgically created ovine MMC model in future investigations 10.

## Conclusion

Injected hAFSCs migrate to the lesion and cover the exposed spinal cord during fetal MMC. They also produce HGF to protect neural elements and promote neural regeneration. Fetal MMC can be diagnosed during an early stage of pregnancy, which means that we can isolate hAFSCs from those patients and use them for in utero therapy. In conclusion, the intra‐amniotic administration of hAFSCs could represent a novel strategy to treat fetal MMC.

## Author Contributions

Y.A.: conception and design, provision of study material or patients, collection and/or assembly of data, data analysis and interpretation, manuscript writing, final approval of manuscript; D.O., H.O., M.T.: conception and design, administrative support, collection and/or assembly of data, data analysis and interpretation, manuscript writing, final approval of manuscript; H.M.: conception and design, collection and/or assembly of data, data analysis and interpretation, manuscript writing, final approval of manuscript; Y.S., T.O.: provision of study material or patients, collection and/or assembly of data, data analysis and interpretation, final approval of manuscript; M.F.: provision of study material or patients, data analysis and interpretation, final approval of manuscript; S.I., K.M.: administrative support, data analysis and interpretation, final approval of manuscript.

## Disclosure of Potential Conflicts of Interest

H.O. is a founding scientist and a paid Scientific Advisor of SanBio Co, Ltd. and K Pharma, Inc. The other authors indicated no potential conflicts of interest.

## Supporting information


**Supplementary Figure S1 Intra‐amniotic cavity injection and phenotypic characterization of retinoic acid (RA)‐induced rat myelomeningocele (MMC) model.**
(A) Representative images of rat uterus (left) with fetuses injected with human amniotic fluid stem cells (hAFSCs) (suspended in PBS [arrow]) and PBS alone (arrowhead). Gross view of intra‐amniotic injection via the ventral aspect of the fetus (right). (B) Representative images of RA‐induced abnormalities in rat fetuses, including normal, spina bifida occulta, MMC (spina bifida aperta), sirenomelia, and gastroschisis (Scale bars, 500 μm). (C) Fetal abnormalities depending on the timing of RA administration on E10 as follows: 0:00 a.m. (group 1), 6:00 a.m. (group 2), 0:00 p.m. (group 3), and 6:00 p.m. (group 4).

**Supplementary Figure S2 Culture, surface marker expression, and differentiation potential of human amniotic fluid stem cells (hAFSCs).**
(A) Representative images of anti‐CD117 immunocytochemistry for human amniotic fluid cells (Scale bars, 100 μm). (B) Macrograph images showing the morphology of hAFSCs (Scale bars, 100 μm). (C) Flow cytometric analysis of surface marker expression on hAFSCs. Mesenchymal markers (CD29, CD44, CD73, CD90, CD105) were positive and hematological markers (CD14, CD34, HLA‐DR) were negative. (D–F) Representative microscopic images of differentiated hAFSCs. The cells were cultured with adipogenic, osteogenic, or chondrogenic differentiation medium for appropriate times, which was assessed by Oil red O, Alizarin red, or Alcian blue staining, respectively (scale bars, 50 μm).

**Supplementary Figure S3 Human amniotic fluid stem cells (hAFSCs) ‐ treatment increases tubulin‐βIII expression in bladder smooth muscle.**
Representative images of (A) HE staining, (B) α‐SMA immunostaining, (C) Tubulin‐βIII immunostaining of bladder in retinoic acid (RA) and RA + hAFSC group (scale bars, 100 μm).

**Supplementary Table S1** List of antibodies used for flow cytometry
**Supplementary Table S2**. List of antibodies used for immunohistochemistry
**Supplementary Table S3**. List of primer sequences used for qRT‐PCR

## Data Availability

The data that support the findings of this study are available from the corresponding author upon reasonable request.
