# Supplementary figures and images for: In Utero Amniotic Fluid Stem Cell Therapy Protects Against Myelomeningocele via Spinal Cord Coverage and Hepatocyte Growth Factor Secretion
Source: Stem Cells Transl Med. 2019 Aug 13;8(11):1170–9. doi: 10.1002/sctm.19-0002 (PMC6811697; doi:10.1002/sctm.19-0002)

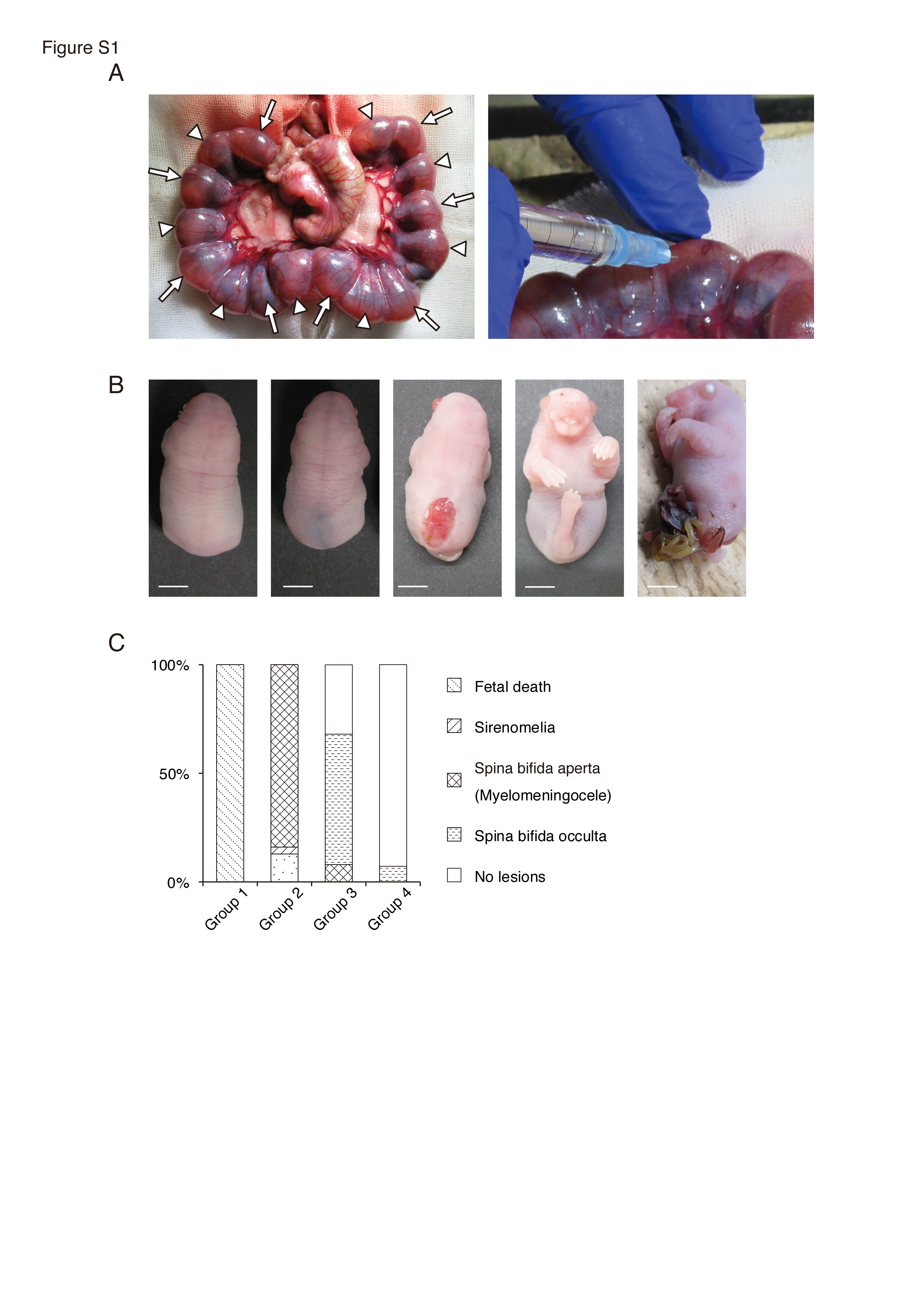

Supplement: Supplementary file 1 — Supplementary Figure S1 Intra‐amniotic cavity injection and phenotypic characterization of retinoic acid (RA)‐induced rat myelomeningocele (MMC) model. (A) Representative images of rat uterus (left) with fetuses injected with human amniotic fluid stem cells (hAFSCs) (suspended in PBS [arrow]) and PBS alone (arrowhead). Gross view of intra‐amniotic injection via the ventral aspect of the fetus (right). (B) Representative images of RA‐induced abnormalities in rat fetuses, including normal, spina bifida occulta, MMC (spina bifida aperta), sirenomelia, and gastroschisis (Scale bars, 500 μm). (C) Fetal abnormalities depending on the timing of RA administration on E10 as follows: 0:00 a.m. (group 1), 6:00 a.m. (group 2), 0:00 p.m. (group 3), and 6:00 p.m. (group 4). [file SCT3-8-1170-s001.tif]

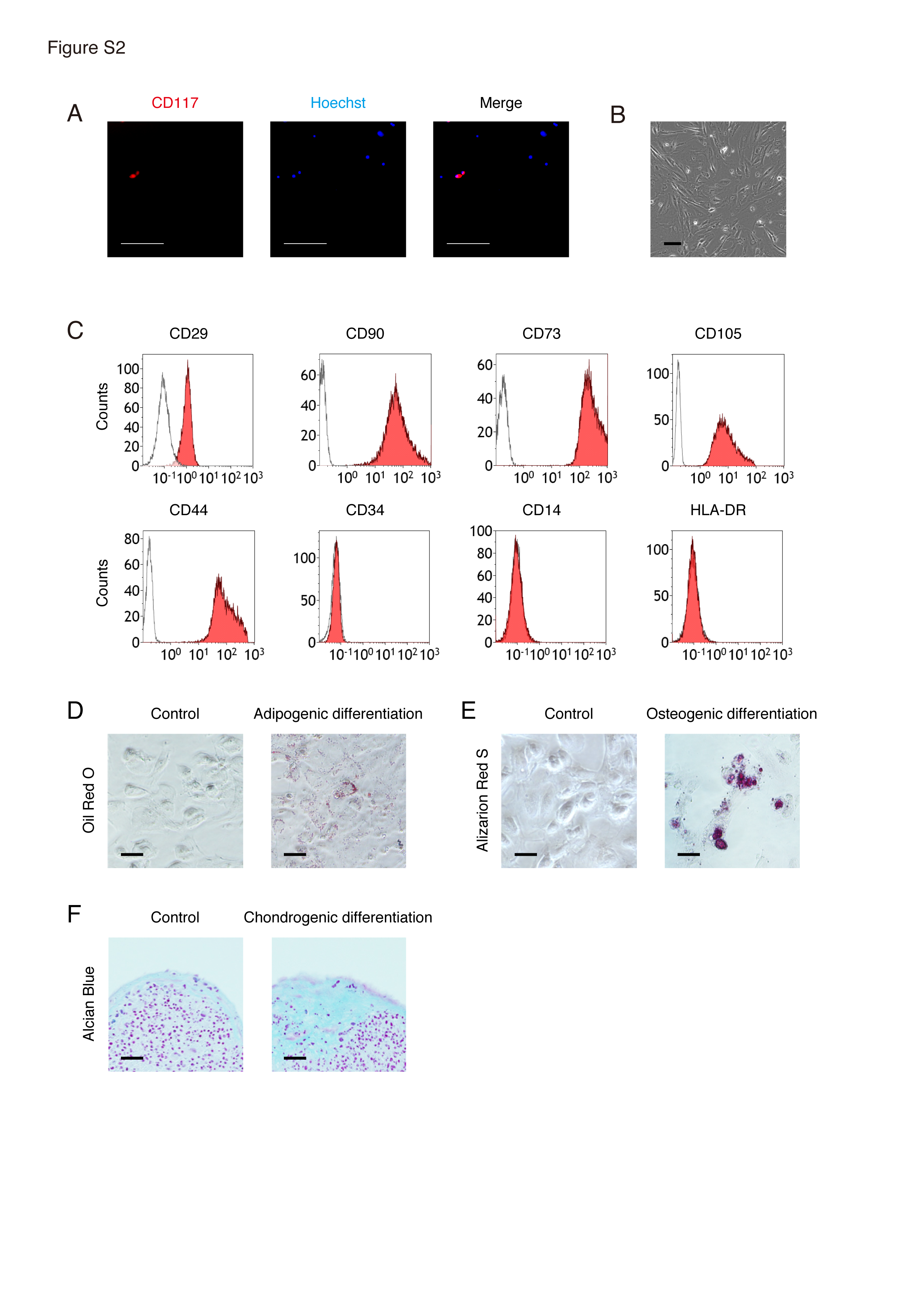

Supplement: Supplementary file 2 — Supplementary Figure S2 Culture, surface marker expression, and differentiation potential of human amniotic fluid stem cells (hAFSCs). (A) Representative images of anti‐CD117 immunocytochemistry for human amniotic fluid cells (Scale bars, 100 μm). (B) Macrograph images showing the morphology of hAFSCs (Scale bars, 100 μm). (C) Flow cytometric analysis of surface marker expression on hAFSCs. Mesenchymal markers (CD29, CD44, CD73, CD90, CD105) were positive and hematological markers (CD14, CD34, HLA‐DR) were negative. (D–F) Representative microscopic images of differentiated hAFSCs. The cells were cultured with adipogenic, osteogenic, or chondrogenic differentiation medium for appropriate times, which was assessed by Oil red O, Alizarin red, or Alcian blue staining, respectively (scale bars, 50 μm). [file SCT3-8-1170-s002.tif]

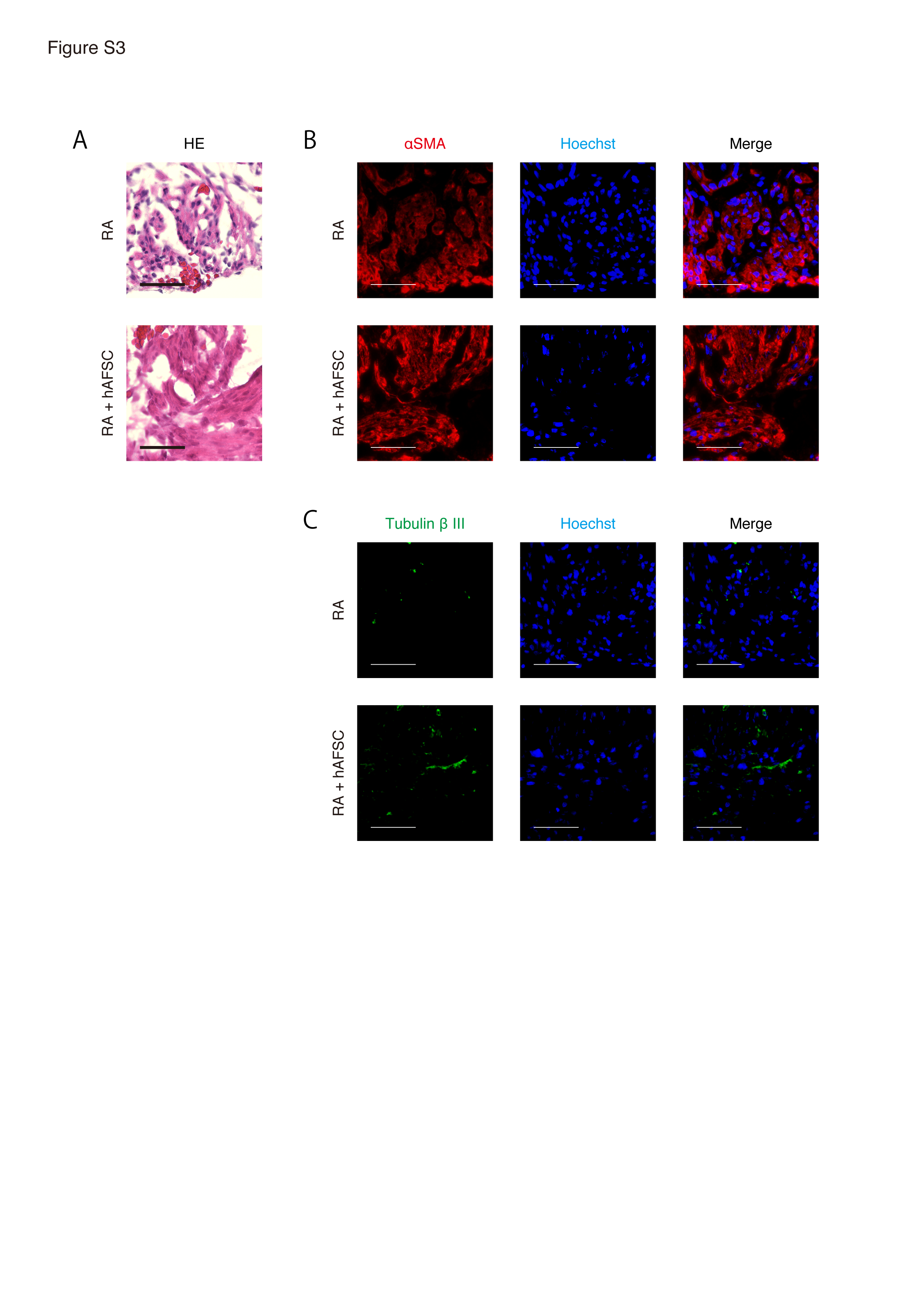

Supplement: Supplementary file 3 — Supplementary Figure S3 Human amniotic fluid stem cells (hAFSCs) ‐ treatment increases tubulin‐βIII expression in bladder smooth muscle. Representative images of (A) HE staining, (B) α‐SMA immunostaining, (C) Tubulin‐βIII immunostaining of bladder in retinoic acid (RA) and RA + hAFSC group (scale bars, 100 μm). [file SCT3-8-1170-s003.tif]
