# Supplementary material for: In Utero Amniotic Fluid Stem Cell Therapy Protects Against Myelomeningocele via Spinal Cord Coverage and Hepatocyte Growth Factor Secretion
Source: Stem Cells Transl Med. 2019 Aug 13;8(11):1170–9. doi: 10.1002/sctm.19-0002 (PMC6811697; doi:10.1002/sctm.19-0002)
Supplement: Supplementary file 4 — Supplementary Table S1 List of antibodies used for flow cytometry Supplementary Table S2. List of antibodies used for immunohistochemistry Supplementary Table S3. List of primer sequences used for qRT‐PCR [file SCT3-8-1170-s004.docx]

| Supplementary Table S1. List of antibodies used for flow cytometry | | | |
| --- | --- | --- | --- |
| Antigen | Clone | Concentration | Source |
| CD14 | TUK4 | 10 μL/2.5 × 10^5^ cells | Miltenyi Biotec |
| CD29 | AG89 | 20 μL/2.5 × 10^5^ cells | MBL CO., LTD |
| CD34 | 581 | 20 μL/2.5 × 10^5^ cells | BD Bioscience Pharmingen |
| CD44 | 44-26 | 20 μL/2.5 × 10^5^ cells | BioLegend |
| CD73 | AD2 | 20 μL/2.5 × 10^5^ cells | BioLegend |
| CD90 | 5E10 | 20 μL/2.5 × 10^5^ cells | BioLegend |
| CD105 | 43A3 | 20 μL/2.5 × 10^5^ cells | BioLegend |
| CD117 | 104D2 | 20 μL/2.5 × 10^5^ cells | BioLegend |
| HLA-DR | B8.12.2 | 20 μL/2.5 × 10^5^ cells | BioLegend |

| Supplementary Table S2. List of antibodies used for immunohistochemistry | | | | | |
| --- | --- | --- | --- | --- | --- |
| Antigen | Host | Type | Dilution | Code | Source |
| Tubulin beta III | Mouse | Monoclonal | 1:100 | ab78078 | Abcam |
| GFAP | Rabbit | Polyclonal | No diluent | IS524 | DAKO |
| STEM121 | Mouse | Monoclonal | 1:500 | Y40410 | TaKaRa |
| Broad-spectrum cytokeratin  (wide CK) | Rabbit | Polyclonal | 1:500 | ab9377 | Abcam |
| CXCL12 | Mouse | Monoclonal | 1:100 | sc-74271 | Santa Cruz |
| HGF | Rabbit | Polyclonal | 1:500 | ab83760 | Abcam |
| c-Met | Rabbit | Polyclonal | 1:200 | bs-0668R | Bioss |
| p-Met | Rabbit | Polyclonal | 1:50 | AF2480-SP | R&D |

| Supplementary Table S3. List of primer sequences used for qRT-PCR | | |
| --- | --- | --- |
| Gene | Forward primer (5′-3′) | Reverse primer (5′-3′) |
| *GAPDH* | CAGCCTCGTCTCATAGACAAGATG | AAGGCAGCCCTGGTAACCA |
| *MCP-1* | AGCCAGATGCAGTTAATGCCC | ACACCTGCTGCTGGTGATTCTC |
| *TNFα* | ATGTGGAACTGGCAGAGGAG | ACGAGCAGGAATGAGAAGAGG |
| *IL-1β* | CACCTCTCAAGCAGAGCACAG | GGGTTCCATGGTGAAGTCAAC |
| *COX-1* | GCCTCGACCACTACCAATGT | AGGTGGCATTCACAAACTCC |
| *COX-2* | TCAAGACAGATCAGAAGCGA | TACCTGAGTGTCTTTGATTG |
| *IL-6* | TCAACTCCATCTGCCCTTCAG | AAGGCAACTGGCTGGAAGTCT |
| *IL-10* | AGAAGCTGAAGACCCTCTGGATAC | GCTCCACTGCCTTGCTTTTATT |
| *HGF* | AGCAATGTTGACTTCACTGACA | AAGACCAACTATATTGCTGTGGA |
| *BDNF* | TGTCCGAGGTGGTAGTACTTCATC | CATGCAACCGAAGTATGAAATAACC |
| *NGF* | ATCGCTCTCCTTCACAGAGTTT | TGTACGGTTCTGCCTGTACG |
| *SDF-1* | TGCGTCCACGAGCTGTTTAC | CCCAAGGGAGTGTCAGGTAGAG |
| *CXCR4* | CACTTCAGATAACTACACCG | ATCCAGACGCCAACATAGAC |
